# spaTransfer: transfer learning for single-cell and spatial transcriptomics data using non-negative matrix factorization

**DOI:** 10.64898/2025.12.12.694021

**Published:** 2026-10-24

**Authors:** Cindy Fang, Kelsey D. Montgomery, Sarah E. Maguire, Anthony D. Ramnauth, Boyi Guo, Ryan Miller, Joel E. Kleinman, Thomas M. Hyde, Keri Martinowich, Kristen R. Maynard, Stephanie C. Page, Stephanie C. Hicks

## Abstract

Recent advances in spatially-resolved transcriptomics have enabled profiling of gene expression in a spatial context, which has led to the generation of large-scale single-cell and spatial atlases with computationally-derived cell type or spatial domain labels. An increasingly important task with these data has become the transfer of cell type or spatial domain annotations from a given reference (or source) atlas into a new target tissue or sample. The reference and target datasets could be at different resolutions or measured on different experimental platforms. Here, we present a method to perform cross-platform transfer learning that takes as input single-cell or spatial domain labels from a reference atlas or dataset and transfers the labels to a target dataset at a similar or different resolution. Specifically, we use non-negative matrix factorization (NMF) on the reference data to identify factors associated with labels of interest and project these factors into the target dataset to label each new observation. We use a multinomial model with the factors as covariates and labels as the response to predict labels in the target dataset. In contrast to existing approaches, the advantage of our approach is interpretability, without compromising on accuracy. We demonstrate the performance of the method in two human brain tissues and show that our model identifies spatially coherent domains in the target datasets with concordance of marker gene expression. We implement spaTransfer in open-source software as an R package (github.com/cindyfang70/spaTransfer).

